# Gap-Controlled Infrared Absorption Spectroscopy: A Unique Interface-Sensitive Spectroscopy Based on the Combination of Linear Spectroscopy and Multivariate Curve Resolution

**DOI:** 10.1021/acs.analchem.5c02765

**Published:** 2025-09-13

**Authors:** Shoichi Maeda, Shunta Chikami, Subin Song, Maria Vanessa Balois-Oguchi, Airi Katase, Glenn Villena Latag, Takuo Tanaka, Tomohiro Hayashi

**Affiliations:** † Department of Materials Science and Engineering, School of Materials Science and Chemical Technology, 13290 Institute of Science Tokyo, 4259 Nagatsuta-cho, Midori-ku, Yokohama-shi, Kanagawa-ken 226-8502, Japan; ‡ Metaphotonics Research Team, 13593 RIKEN Center for Advanced Photonics, Hirosawa, Wako, Saitama 351-0198, Japan

## Abstract

We present an interface-sensitive spectroscopy method that integrates attenuated total reflection infrared absorption (ATR-IR) spectroscopy, a distance control system, and multivariate curve resolution (MCR). In this approach, we adjust the distance between the sample and the ATR prism while collecting a series of spectra that reflect various contributions from both bulk and interfacial regions. Subsequently, we utilize MCR to extract the spectral components specific to the interfacial region. Here, we validate this method through the analysis of interfacial water adjacent to self-assembled monolayers (SAMs), quartz, polymers, and polymer brush films. Our findings are compared with results from other interface-sensitive spectroscopic techniques, confirming the interface sensitivity of our approach. This method does not necessitate surface enhancement or nonlinear optical effects and imposes virtually no restrictions on the types of samples suitable for analysis. Furthermore, it allows us to assess the thickness of the interfacial region using a spectral component distinct from the bulk region, revealing insights into the relationship between the interfacial behavior of molecules and related phenomena. Additionally, this method can be seamlessly integrated into standard ATR-IR spectroscopes, offering a straightforward solution for interface-sensitive spectroscopy.

## Graphical abstract

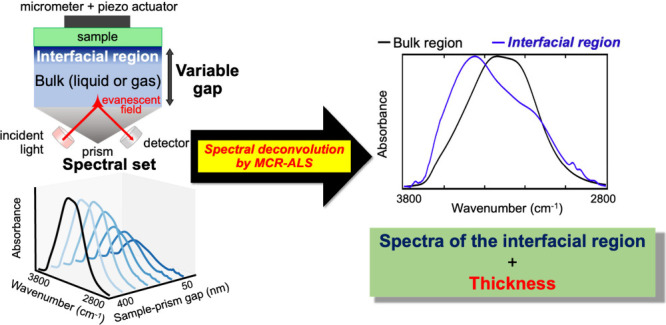

## Introduction

Surface and interface phenomena such as chemical reactions, friction, adsorption, desorption, etc., are governed by microscopic behavior of the molecules in interfacial regions with a finite thickness.^1^ In the electrochemical process, the orientation of the reactive species and water or organic molecules determine the potential field in the interfacial region, and they affect the reaction selectivity and mass transport.^2,3^ The molecules at the interface strongly govern friction and adhesion.^4,5^ In biological systems, the interfacial water plays a crucial role in inter- and intramolecular interactions, governing molecular recognition, enzymic reaction, protein folding, etc.^6,7^

A major goal in this field is to understand how molecular behavior at interfaces governs interfacial phenomena. For this purpose, various surface- or interface-sensitive analytical approaches have been developed. Nonlinear optical spectroscopies such as sum-frequency generation (SFG) and second harmonic generation (SHG) are powerful surface- and interface-sensitive techniques for exploring interfaces where the inversion symmetry is broken.^8–10^ In the cases of well-defined interfaces with a single-crystalline structure, these methods provide accurate information on the orientation of the interfacial molecules and their interactions with a spatial resolution at the submolecular level.^10–12^ On the other hand, these methods encounter challenges in investigating the entire interface region with a finite thickness (up to several nm in single-component liquids and several tens of nm for multicomponent liquids) because interfacial phenomena are often governed not only by the molecules adjacent to material surfaces but also by the molecules in the interfacial region.^7,13,14^

Spectroscopic methods that utilize a surface-enhancement effect, such as surface-enhanced Raman scattering spectroscopy (SERS) and surface-enhanced infrared absorption spectroscopy (SEIRAS), are also interface-selective techniques. These methods employ the locally enhanced electromagnetic field generated by plasmonic resonance, with the field’s distribution depending on the metallic nanostructures. In these instances, we cannot ascertain whether the field fully covers the interfacial region or if the signal from the bulk region is included. Generally, it is challenging to precisely control the distribution of the sizes and shapes of nanostructures fabricated by evaporation or sputtering methods. This results in the difficulty in the evaluation of the measuring regions of SERS and SEIRA.

As discussed, there remains a lack of methods to selectively extract spectral information from interfacial regions, where molecular behavior differs from the bulk. Recently, multivariate analysis methods have been used to extract spectral components from a set of spectra measured with various parameters.^15–17^ In these works, the systems were modulated by changing temperature, concentration, etc. Then, the sets of spectra are acquired, and a spectral component of interest can be obtained through multivariate curve resolution (MCR). Here, we propose a new interface-sensitive method to observe the vibrational spectra of the interfacial region, which are different from those of the bulk region. We investigated various surfaces for the demonstration, including self-assembled monolayers (SAMs), single-crystal quartz, polymer, and polymer brush films. The SAMs and quartz surfaces have been investigated by SFG by several groups. As for the polymer and polymer brush films, they attract much interest in biosensing, tribology, coatings, etc. We compare our results with those obtained using other interface-sensitive techniques to validate our methods.

## Basic Principle of Gap-Controlled ATR-IR Spectroscopy

Figure 1 illustrates the basic concept. This method places a sample at a certain distance (50 to 1000 μm) above the attenuated total reflection infrared (ATR-IR) prism, facing the prism. The gap distance between the sample and the ATR-IR prism is controllable. In this configuration, water is sandwiched between the prism and the sample, creating a system that has three components: the interfacial water of the sample (*C*_s_), the bulk water (*C*_b_), and the interfacial water of the prism (*C*_p_). When we change the distance between the sample and the prism, *C*_s_ and *C*_b_ change, while *C*_p_ remains constant. Consequently, *C*_p_ is canceled by subtracting the spectra measured at different gaps. From the set of spectra containing *C*_s_ and *C*_b_ with various ratios, we can separate the spectral set into the two spectral components of the interfacial region near the sample and bulk (known) using MCR.

**Figure 1:**
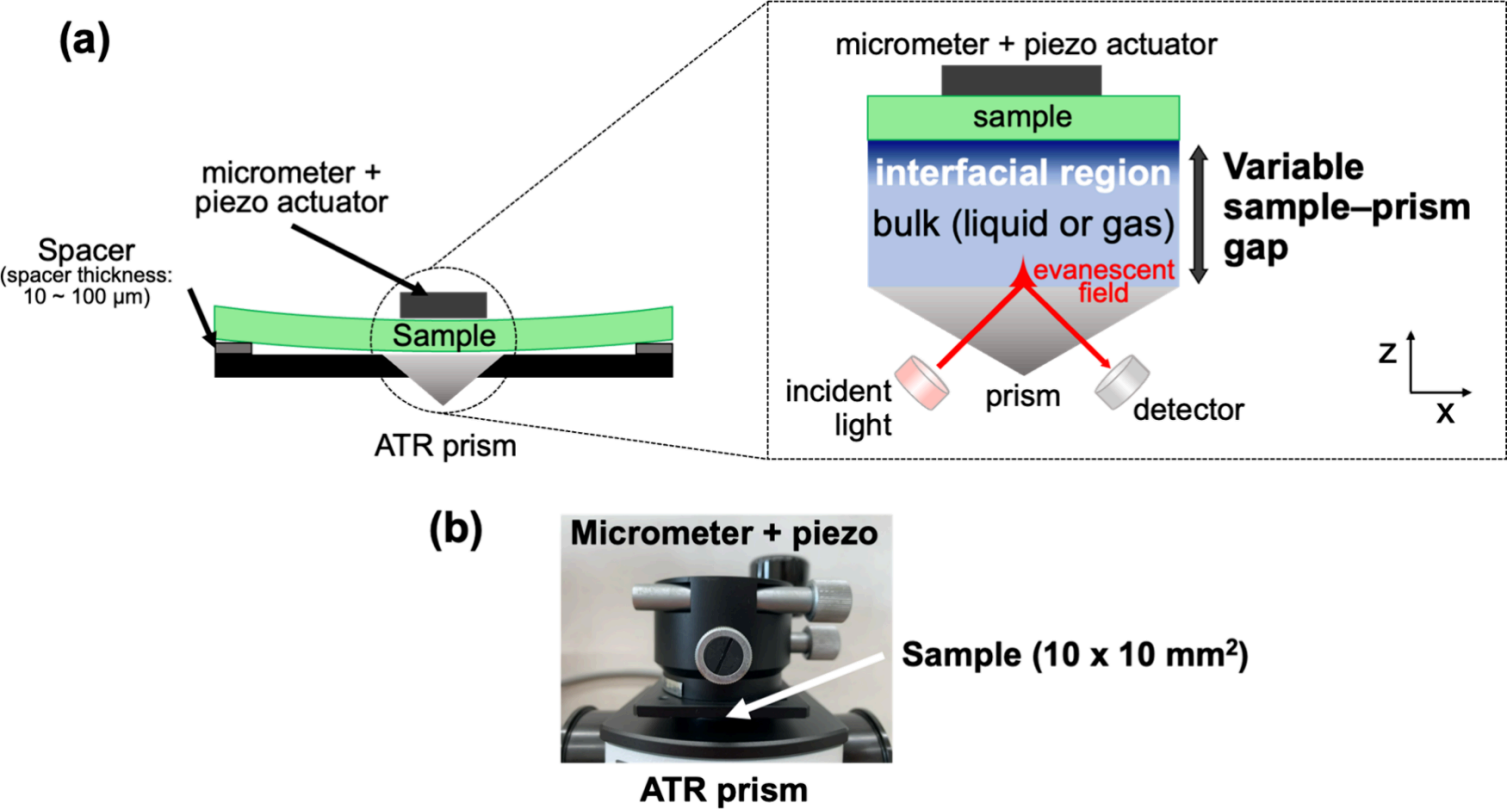
(a) Illustration of gap-controlled ATR-IR spectroscopy. (b) Side-view photo of the experimental setup.

## Methodology

The experimental setup consists of a commercial ATR-IR spectrometer equipped with a diamond ATR prism (FT/IR-6600, JASCO Inc., Tokyo, Japan) and a control system for the sample-prism distance, as shown in Figure 1. The system includes a sample and a bulk region, which can be either liquid or gas. Additionally, there is an interfacial region between the sample and the bulk region, which is the primary focus of this study. We utilized a micrometer and a piezoelectric actuator to precisely control the sample–prism gap within the submicron range.

In ATR-IR spectroscopy, the intensity of the electromagnetic field of the evanescent wave, *E*_*z*_, exponentially decreases as the distance from the ATR prism, *z*, decreases.^18^ The ATR-IR signal at each distance calculated by the square of the intensity of the evanescent wave, can be expressed by eq 1 and is illustrated in Figure 2 (a):

1 $${\left|E_{z}\right|}^{2}={\left|E_{0}\exp\left(-\frac{z}{d_{p}}\right)\right|}^{2}={\left|E_{0}\right|}^{2}\exp\left(-\frac{2z}{d_{p}}\right)$$

where *E*_0_ represents the electric field of the evanescent wave when z = 0, and *d*_p_ denotes the decay length of the evanescent wave, which is given by eq 2:

2 $$d_{p}=\frac{\lambda }{2\pi n_{1}\sqrt{\sin^{2}\theta -{\left(n_{2}/n_{1}\right)}^{2}}}$$

where *n*_1_, *n*_2_, λ, and θ represent the refractive indices of an ATR prism and a sample, the wavelength of the infrared light, and the incident angle, respectively. When utilizing an ATR diamond prism with a refractive index of *n*_1_ = 2.42, water with a refractive index of *n*_2_ = 1.33, a wavelength of λ = 1/3300 cm (3300 cm^–1^ is the midpoint of the wave number range from 3800 to 2800 cm^–1^), and an incident angle of θ = 45°, the value of the decay length, *d*_p_ is calculated to be 448 nm.

**Figure 2:**
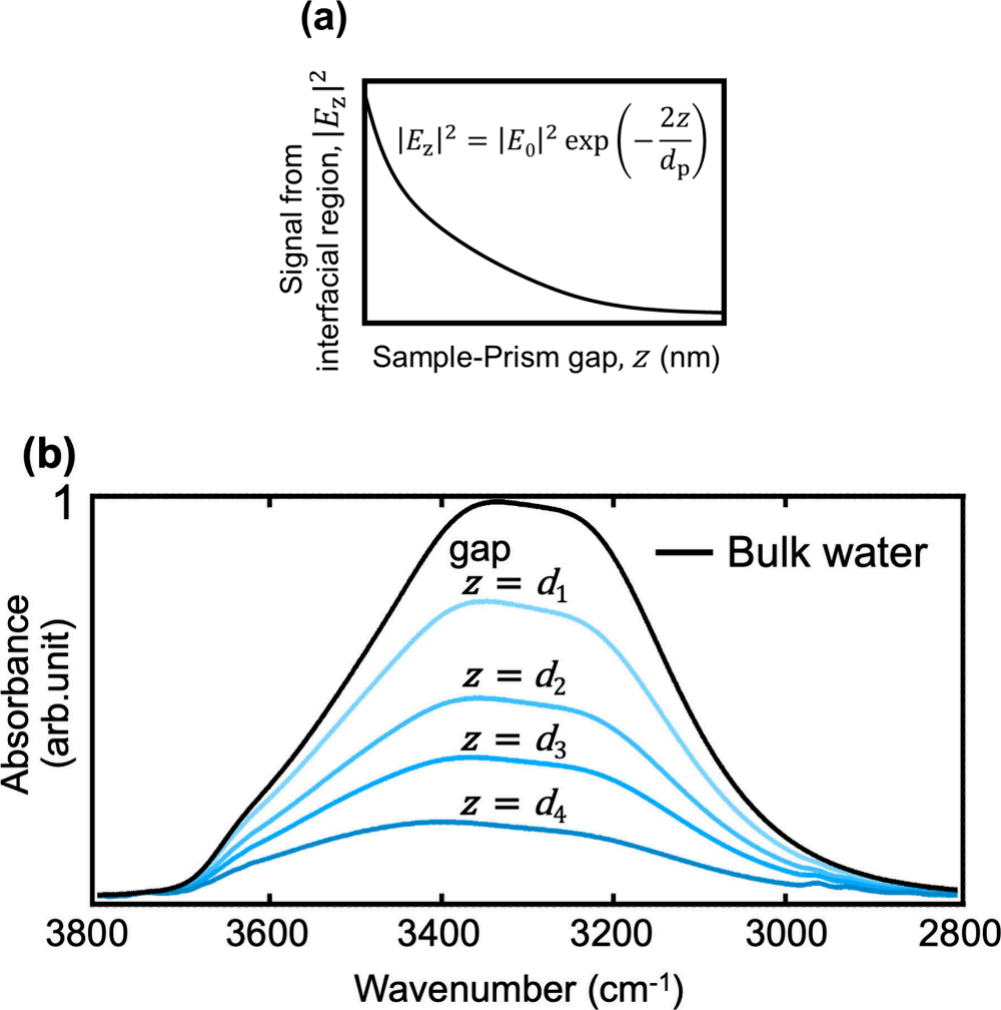
(a) Theoretical curve of the evanescent field intensity (penetration depth of 448 nm) and (b) set of ATR spectra of water on C8 SAM in the OH stretching region. (*d*_1_ > *d*_2_ > *d*_3_ > *d*_4_). The thicknesses, *d*_1_, *d*_2_, *d*_3_, and *d*_4_, are 325, 170, 107, and 34 nm, respectively.

The intensity of an ATR-IR spectrum, *I*, is obtained by integrating the ATR-IR signal at each sample-prism distance using eq 1, as shown in eq 3:

3 $$I=\int_{0}^{\infty }{\left|E_{z}\right|}^{2}dz=\int_{0}^{\infty }\left\{{\left|E_{0}\right|}^{2}\exp\left(-\frac{2z}{d_{p}}\right)\right\}\mathrm{dz}=1-\exp\left(-\frac{2z}{d_{p}}\right)$$

By approaching a sample close to the prism, the signal from the interfacial region between the sample and bulk increases, as shown in Figure 2(a). Applying this principle, we utilized a micrometer and a piezoelectric actuator to gradually reduce the sample-prism gap within a range of a few nanometers to obtain a set of ATR spectra that contains several spectra with gradually increasing signals from the interfacial region.

Figure 2(b) shows the ATR spectra of water obtained by the gap-controlled ATR-IR spectroscopy, including signals from bulk water and interfacial water. Here, a self-assembled monolayer (SAM) with terminal groups of CH_3_, referred to as C8 SAM, was used as the sample, and pure water as the bulk water. The spectra were processed in Python (version 3.8.5) using the Savitzky-Golay method for smoothing and Pybaselines for baseline correction. It should be noted that the smoothing process is not necessary when the data acquisition time is long enough to get a sufficient signal-to-noise ratio. The wavenumber range from 3800 to 3000 cm^–1^ reflects the O–H stretching modes of water molecules. The peaks attributed to the CH stretching modes of the molecules constituting the C8 SAM were observed below a wavenumber of 3000 cm^–1^. The intensity of the peaks increases as the gap distance becomes smaller, evidencing that the sample surface approaches the prism with the gap-controlling system.

The set of spectra in Figure 2(b) shows gradual changes in both intensity and spectral shape as the sample-prism gap decreases. The decrease in spectral intensity is attributed to the reduction of bulk water, facilitated by the gap-controlled technique. The changes in spectral shape indicate an increase in the signal of interfacial water. A spectrum of bulk water was also obtained and included in the spectra to provide initial estimates needed for the following spectral deconvolution. Subsequently, spectral deconvolution was performed using the multivariate curve resolution—alternating least-squares (MCR-ALS) method to extract the pure spectral component of interfacial water. We can also evaluate the thickness of the interfacial water from the concentration (C) of the spectra (eq S7 in Supporting Information).

In the Supporting Information, we describe the following details: spectral deconvolution using MCR-ALS, the calculation of thicknesses of interfacial regions, a finite-difference time-domain (FDTD) calculation evaluating the electromagnetic field in the gap, and validation of the sample-and-prism parallelism

## Results and Discussion

### PDMS–Water interface

Poly­(dimethylsiloxane) (PDMS) is a typical hydrophobic material (water contact angle is 105–115°), and its interfacial water was investigated by SFG. Figure 3 compares the ATR-IR spectra of bulk water, the spectra of the interfacial water obtained by our method and SFG.^19^ As seen, the two spectra of the interfacial water differ from that of bulk water. In particular, the spectra obtained by our method and SFG exhibit several sharp peaks in the 3600 to 3800 cm^–1^ region. These peaks are assigned to the OH stretching band of OH bonds without forming hydrogen bonding (so-called isolated OH bond), typically observed for water in the gas phase. Compared with bulk water, another prominent feature of the interfacial water’s spectra is the strong signal between 3200 and 3100 cm-1. A large part of this region is assigned to the symmetric stretching band of the water with four hydrogen bonds (ice-like). This feature is observed for hydrophobic-water interfaces.^20–23^ Water molecules near a hydrophobic surface cannot form hydrogen bonds with the surface and maximize the number of hydrogen bonds with adjacent molecules (so-called ice-berg formation).

**Figure 3:**
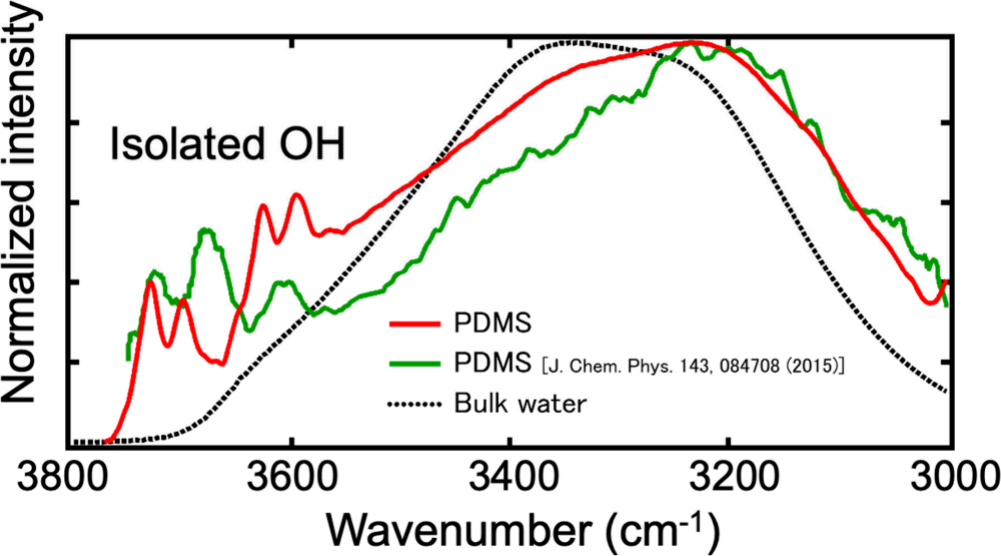
ATR-IR spectra (corrected with the penetration depth) of bulk water, SFG, and our spectra of a water–PDMS interface. The SFG result was reproduced from ref 17.

Previous X-ray reflectivity measurements, the grand canonical Monte Carlo simulations, and molecular dynamics revealed that a density depletion layer with a thickness of approximately 0.5 nm exists near hydrophobic surfaces. They also reported a region where water molecules are highly oriented, above the depletion layer.^24–26^ The thickness of the region with the oriented water molecules is about 1 nm, which is in good agreement with our results (1.5 (±0.2) nm). The simulation indicated a density depletion layer and a local increase in the hydration bond number at the interface. The SFG and our results support this finding. These features demonstrate the interface selectivity of our method. Next, we will discuss the results using model organic and inorganic surfaces to further verify this method’s validity.

### Interface between Water and Self-Assembled Monolayers

Self-assembled monolayers (SAMs) of alkanethiols on Au(111) are ideal model organic surfaces because of their ultraflat and well-defined structures.^27–29^ By the selecting terminal groups of the alkanethiols constituting SAMs, we can create surfaces with a variety of physicochemical properties. Here we employed three types of SAMs, i.e., hydrophobic (C8: methyl-terminated), hydrophilic (OH: hydroxyl-terminated), and hydrophilic-and-antifouling (EG3-OH: oligo­(ethylene glycol)-terminated).^30–32^

Figure 4(a) and (b) compares the results of spectra measured by our method and surface-enhanced infrared absorption spectroscopy (SEIRAS).^33^ We found that both methods provided similar results in terms of spectral shape, highlighting the interface selectivity of this method. Next, we discuss the details of the spectra obtained with the three SAMs. The main peaks at around at 3450 cm^–1^ are assigned to the OH stretching modes of weakly bound water molecules (two states model)^34^ or OH stretching modes of water molecules with the DA (one donor and one acceptor are involved in the hydrogen bonds) and DDA (two donors and one acceptor are involved in the hydrogen bonds) hydrogen bonding configurations.^35^ This indicates that the hydrogen bonding states of water in the vicinity of the SAMs are different from those of bulk water.

**Figure 4:**
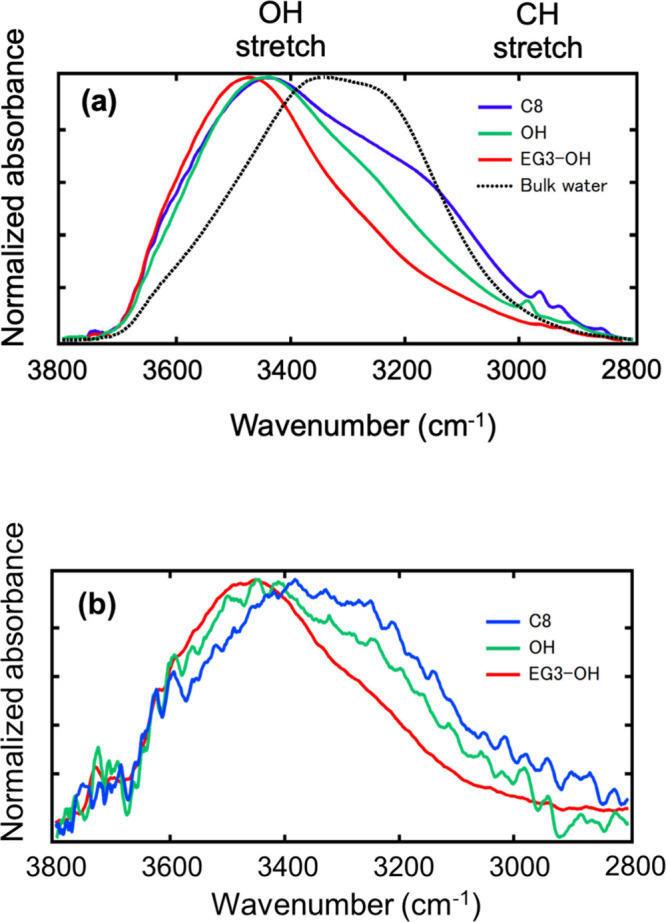
ATR-IR spectra of interfacial water of C8, OH, and EG3-OH SAMs in the OH stretching region obtained by (a) our technique and (b) SEIRAS.^33^

The most prominent spectral difference arises between 3200 and 3100 cm^–1^, which is assigned to the OH stretching mode of a water molecule that forms four hydrogen bonds. The water near the C8 SAM showed stronger intensity than that near the OH and EG3-OH SAMs. In particular, the spectra for C8 SAM displayed a stronger signal than that for bulk water, indicating that the strength of the hydrogen bonds between the interfacial water molecules became stronger, a typical hydrophobic effect as mentioned before. In contrast, the water near the OH and EG3-OH SAMs exhibited weaker intensity at around 3100 cm^–1^. These SAMs provide hydrogen bonding sites to the interfacial water, resulting in the absence of the hydrophobic feature observed with PDMS and CB SAM. When comparing OH and EG3-OH SAMs, the spectrum observed for EG3-OH SAM is sharper than that observed for OH SAM, indicating that the orientation of the vicinal water of EG3-OH SAM is more uniform than that of OH. This can be rationalized by the fact that the EG3-OH molecule has more hydrogen bonding sites than the OH molecule.

The comparison of the thickness of interfacial water provides a deeper insight into the correlation between molecular behavior and interfacial phenomena. The thickness of the interfacial water for the C8 SAM is 1.1 (±0.2) nm, almost equal to the thickness observed for PDMS. As for the hydrophilic SAMs, the EG3-OH possesses a thicker hydration layer than the OH SAM (4.3 (±0.4) and 2.3 (±0.4) nm), as shown in Figure 5. We believe that the larger number of hydrogen bonding sites in the EG3-OH molecules is the reason for the difference.

**Figure 5:**
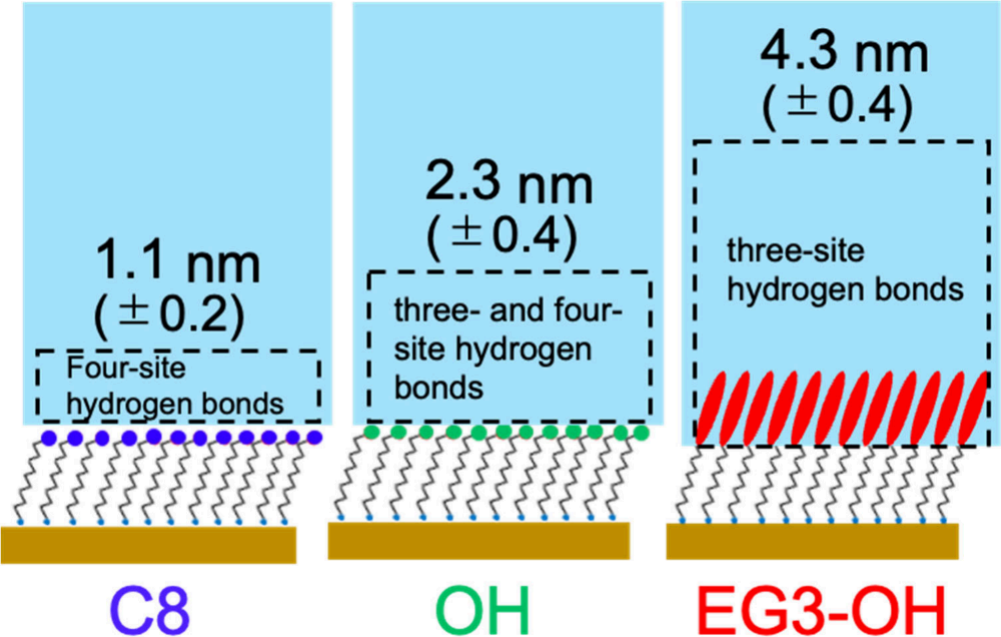
Thicknesses of interfacial water of C8, OH, and EG3-OH SAMs obtained by MCR-ALS analysis.

Interestingly, the thickness obtained with this method is almost equal to that observed by the surface force measurement using atomic force microscopy (AFM). Our previous works clarified that the interfacial water of EG3-OH plays the role of a physical barrier to deter proteins and cells from adsorption or adhesion.^30–32^ Our results confirm that we successfully measured the hydrogen bonding states of the water barrier, demonstrating the effectiveness of our method to connect the behavior of interfacial water and materials’ functions.

Finally, we discuss the difference between the results of hydrophobic PDMS and C8 SAM. The sharp isolated OH stretching peaks (3600 to 3800 cm^–1^) were not observed for the hydrophobic C8 SAM, although the peaks were observed for PDMS. We consider that the difference in the spectra originates from the difference in the wetting manner in the microscopic view. First, the chemical structures are different. The topmost surface of the C8-SAM is covered with methyl groups, and water molecules cannot penetrate into the SAMs. In contrast, the PDMS surface consists of cross-linked dimethylsiloxane, and water can be trapped in the microscopic cavities in PDMS. Therefore, the interfacial water of PDMS shows the isolated OH stretching modes.

### Hydration Structures of Quartz at Various pH

A quartz surface is one of the most studied single-crystal inorganic solid surfaces by various experimental techniques.^11,36–38^ The surface is covered with silanol groups in air, and these groups are protonated or deprotonated depending on pH values, generating surface charges. Here, we investigated the effect of the surface charges on the interfacial water using quartz surfaces. Figure 6(a) shows the spectrum of bulk water and the spectra of interfacial water of the quartz at three different pH values in water. The three spectra shown in Figure 6 resemble those measured with hydrophilic OH and EG3-OH SAMs, and the spectra did not show the feature observed for the hydrophobic surfaces (PDMS and C8 SAM). The shapes of the three spectra are similar, but the intensity between 3100 and 3000 cm^–1^ becomes stronger as the negative surface charges increase (at higher pH values). Shen and Ostroverkhov reported SFG results, in which the SFG intensity at 3200 cm^–1^ is strong at high pH.^39,40^ They assigned the peak at 3200 cm^–1^ as the symmetric OH stretching band of water in an ice-like structure. Also, for this case, we confirmed the agreement in interpreting the molecular behavior of the interfacial water with SFG.

**Figure 6:**
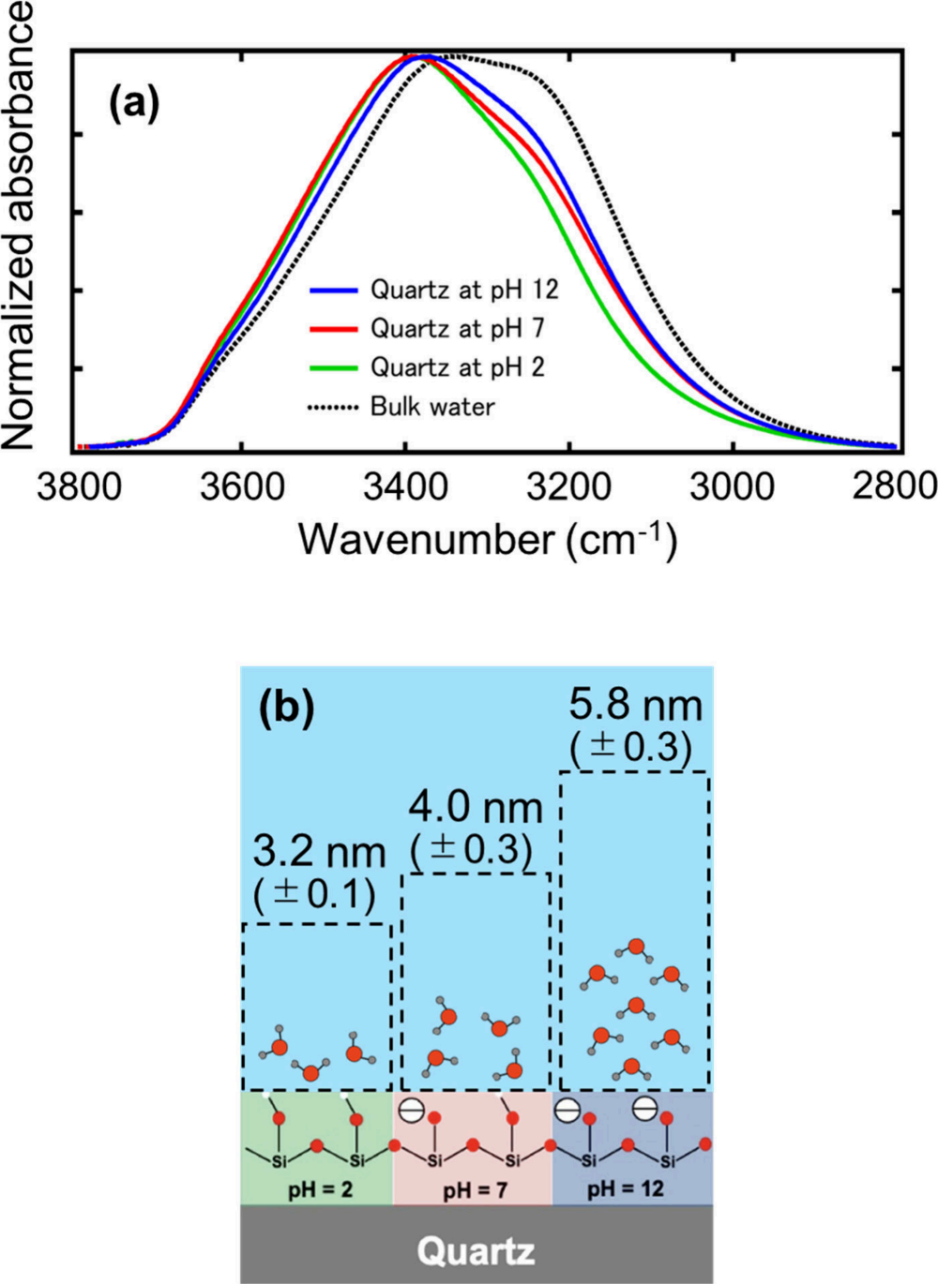
(a) ATR-IR spectra of interfacial water of quartz at three pH values in the OH stretching region obtained by MCR-ALS analysis. (b) Schematic image of hydration structures of quartz at three pH values.

Our result also shows that the thickness of the interfacial water increases with increasing pH values, with respective thicknesses of 3.2 (±0.1), 4.0 (±0.3), and 5.8 (±0.3) nm at pH 2, 7, and 12, respectively (Figure 6 (b)). This result suggests that the density of the negative surface charges governs the ordering of water molecules deeper into bulk water. This feature was also observed in the SFG measurements, in which the SFG signal increased as the pH.^41^

### Hydration Structures of Polymer Brush Films

Polymer brush films are more practical than SAMs regarding chemical and physical stability. The polymer blush films have been used to fabricate surfaces for cell-scaffolding layers, biosensors, etc. Here, we investigate the interfacial water of two representative antifouling polymer brush films, i.e., nonionic poly­(ethylene glycol) (PEG) and zwitterionic poly­(carboxybetaine methacrylate) (PCBMA).

Figure 7 displays the spectra of interfacial water obtained using our method. Comparing the spectra between the PEG and PCBMA films, the spectrum of interfacial water in PEG shows higher intensity in the higher wavenumber range (from 3650 to 3450 cm-1), while PCBMA displays higher intensity in the lower wavenumber range (from 3400 to 3000 cm-1). The primary interaction between PEG and water involves hydrogen bonds between the ether oxygen of PEG and the hydrogen atoms of water molecules (proton-accepting surfaces). This interaction significantly alters the hydrogen bonding network of interfacial water compared to its bulk state, leading to substantial deviations from the spectra of bulk water. This pattern was also observed for EG3-OH SAM (Figure 4). In contrast, the spectra of interfacial water in PCBMA are quite similar to the spectra of bulk water, indicating that PCBMA does not disrupt the native hydrogen bonding states of bulk water. Kitano et al. reported that polymers which do not significantly affect the structure of water on their surfaces, or those that weakly bond with water molecules, exhibit high antifouling properties, aligning with our findings.

**Figure 7:**
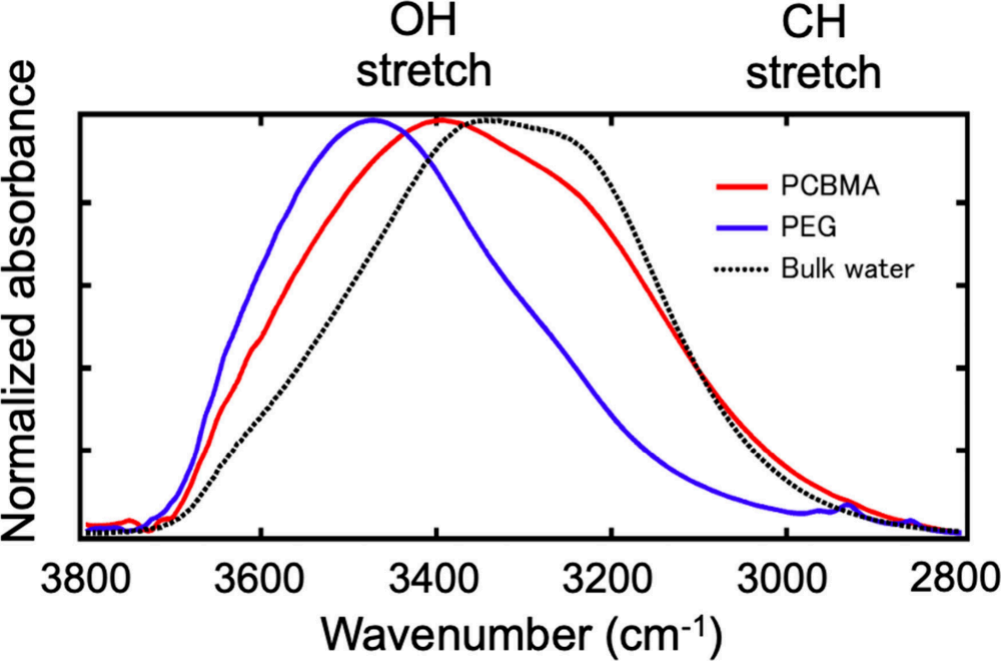
ATR-IR spectra of interfacial water of PEG and PCBMA brush films in the OH stretching region obtained by MCR-ALS analysis.

In the case of polymer brush films, there is no straightforward interface between the films and water. Therefore, it is not easy to evaluate the thickness of the interfacial water, because water molecules penetrate the polymer brushes. In this method, the spectra of interfacial water obtained with the MCR-ALS contain the signals from the molecules constituting the SAMs, because the MLR-ALS separates measured spectra into the spectra of bulk (fixed in the fitting) and interfacial regions. We can acquire a detailed picture of the interface by comparing the relative intensity ratio between water and polymer. Weak and sharp peaks located at 2850–2950 cm^–1^ are assigned as the C–H stretching modes of the polymer chains. Although both polymers contain methylene or methyl groups, the C–H stretching bands are visible only for PEG, indicating that the water molecules and PEG chains are mixed in the interfacial region. On the other hand, the PCBMA shows a weaker CH stretching signal, and the spectral shape for PCBMA is closer to that of bulk water, suggesting that the PCBMA chains immobilizes more interfacial water molecules compared with PEG with keeping the hydrogen bonding state close to the bulk water. This finding is in agreement with the results of SFG.^42^

### Hydration Structures Polystyrene Surface

Polystyrene (PS) is a popular polymeric material in our daily lives, and it is used as a material for culturing dishes for cell experiments in biology. It is well-known that the PS surface, which is originally hydrophobic (water contact angle: WCA is about 90°), is transformed into hydrophilic (20 to 60° in WCA) by UV/ozone or plasma treatments.^43^ This process is a typical way to enhance surface hydrophilicity and cell adhesion. In this process, a part of the polymer chains is converted to hydrophilic functional groups such as hydroxyl, carbonyl, and carboxyl groups. The polymer surface becomes rough with the chemical transformation, forming a soft layer of hydrophilic polymer chains and water.

For untreated PS, the spectral shape is between hydrophobic (C8 SAM) and hydrophilic (OH SAM), which is explained by the WCA of PS. The plasma-treated PS exhibited a sharper spectrum than the untreated PS. This is attributed to the restriction of water orientation by the formed hydrophilic groups. This trend was also observed with the SAMs.

Next, we discuss the intermixing of the polymer chains and water at the interface based on the spectra shown in Figure 8(a). The intensity ratio of water to the CH stretching mode for untreated PS is smaller than that for treated PS. This indicates that the intermixing of the polymer with water is more significant for the treated PS. As shown in the AFM images observed in water (Supporting Information), the PS surface is roughened and the surface Young’s modulus is lower, suggesting that the polymer surface allows water to penetrate into the diffused polymer layer containing the generated hydrophilic functional groups, as illustrated in Figure 8(b). As we demonstrated here, we can obtain a three-dimensional picture of the behavior of molecules at complex interfaces using our method.

**Figure 8:**
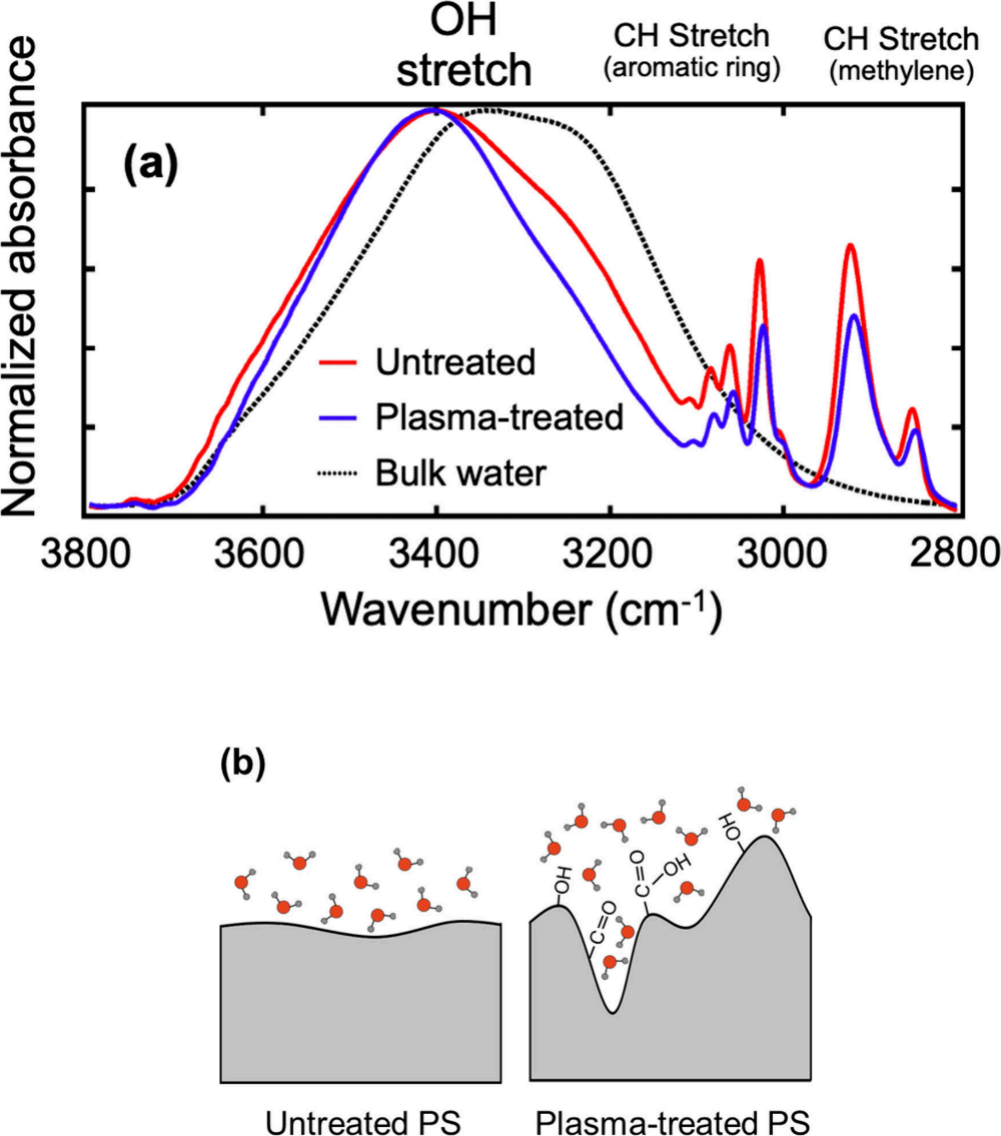
(a) ATR-IR spectra of interfacial water of untreated (in red) and plasma-treated (in blue) polystyrene dishes in the OH stretching region obtained by MCR-ALS analysis. (b) Schematic images of the hydration structures of each polystyrene dish.

It should be noted that this method overestimates the thickness of the interfacial region because the total surface area is larger than that of a flat surface. In this case, we might calibrate the obtained thickness with the total surface area measured using AFM techniques.

### Advantages and Limitations of This Method

A key advantage of this method is its ability to evaluate the thickness of interfacial regions based on distinct spectral features arising from molecular behavior that differs from that of the bulk. Compared with conventional interface-sensitive techniques for investigating interfaces without inversion symmetry using nonlinear optical effects (e.g., SFG and SHG), our method can examine the entire interfacial region, where molecules partake in various interfacial phenomena, with a finite thickness. Combined with the results obtained by SFG or SHG, our method can provide a detailed picture of molecular behavior at interfaces.

Regarding interface-sensitive spectroscopies that utilize the plasmonic enhancement effect (e.g., SEIRA and SERS), the measured region is determined by the distribution of the enhanced electromagnetic field around the nano metallic structures. With this approach, it remains uncertain whether the electromagnetic field encompasses the entire interfacial area and whether a signal from the bulk is included. Additionally, SEIRA and SERS require metallic nanostructures for plasmonic enhancement (typically gold or silver), which limits the materials that can be measured. Conversely, our method imposes no sample limitations. Moreover, perfectly flat surfaces are not necessary because we can deconvolute bulk and interface spectra using MCR. To assess the thickness of the interfacial region, we require calibration to compensate for the increase in surface area using atomic force microscope images. Furthermore, we cannot determine the exact thickness of the interfacial region for samples with diffused interfaces, such as the polymer brush systems presented. In such cases, we need to develop calibration curves based on signals from water and monomers constituting polymers with known concentrations. By analyzing the ratio of the water and polymer signals in the spectra of the interface, we can evaluate the hydration number of each repeating unit in the polymer film.

Another practical advantage of this method is its cost. Our method does not require nonlinear optics, and conventional ATR-IR is easily capable of performing the interface-sensitive analysis by employing a simple gap control system consisting of a spacer and micrometer (piezo actuator and/or mechanical micrometer). Our method is not limited to interface-sensitive vibrational spectroscopy; it can endow any spectroscopic methods using ATR optical configurations with interface sensitivity, including UV–vis absorption spectroscopy, Raman scattering, and fluorescence microscopy. The only requirement is resolution in the signal detection. The signal intensity of the interfacial region is sometimes as low as 1% of the total signal, so accurate extraction of the signal from the interfacial region is necessary.

Finally, we note the limitations of our method. To determine the thicknesses of the interfacial region, we assume a layer with a constant density of bulk water. However, X-ray reflectivity measurements have revealed a depletion region with lower water density (thickness 1 nm), a local enhancement in the density of water near hydrophilic surfaces, and density oscillations in several molecular layers. Therefore, if the thickness of the interfacial region is small (<1 nm), the evaluated thickness needs to be verified using other methods, such as X-ray reflectivity measurements. We conclude that this method will be extremely powerful when combined with X-ray reflectivity measurements (detailed density profile), heterodetection type SFG (exact molecular orientations at the interface), and surface force measurements (force induced by molecules in interfacial regions).

## Concluding Remarks

We proposed a new interface-sensitive vibrational spectroscopy based on a combination of ATR-IR and MCR. In this method, the distance between the ATR prism and the sample was controlled, resulting in a set of spectra that included signals from interfacial and bulk regions with different mixing ratios. We then employed an MCR procedure to extract the spectral components of the interfacial regions along with their thicknesses.

As demonstrations, we measured the IR spectra of water molecules in the vicinity of SAMs with various terminal groups. The shapes of the spectra measured with this method agree well with those obtained from SEIRA, clearly indicating the capability of this method to selectively measure interfaces. Additionally, the thickness of the interfacial water aligns with the thickness measured through surface force measurements. These results suggest that we can connect interfacial behavior and phenomena. Furthermore, the results obtained with the quartz surfaces at different pH levels appear reasonable and correlate well with previous SFG results. Moreover, we demonstrated our method for analyzing surfaces used in practical applications (plasma-treated polystyrene and polymer brush films).

Despite the aforementioned limitations in the results and discussion part, our method allows us to investigate various liquid–solid and gas–solid interfaces in the fields of fundamental surface and interface science, biomaterials, catalysts, batteries, and tribology. We hope that this method reveals the intriguing correlations between the interfacial behaviors of molecules and interfacial phenomena, contributing to the design of functional interfaces in the future.

## Supplementary Material



## Notes

Theoretical details of gap-controlled ATR-IR spectroscopy, materials, and calculations of electromagnetic fields (PDF)
