# Supplementary material for: Gap-Controlled Infrared Absorption Spectroscopy: A Unique Interface-Sensitive Spectroscopy Based on the Combination of Linear Spectroscopy and Multivariate Curve Resolution
Source: Anal Chem. 2025 Sep 13;97(37):20156–63. doi: 10.1021/acs.analchem.5c02765 (PMC12461681; doi:10.1021/acs.analchem.5c02765)
Supplement: Supplementary file 1 [file ac5c02765_si_001.pdf]

[Supporting information]

Gap-controlled infrared absorption spectroscopy: a unique interface-sensitive spectroscopy based on the combination of linear spectroscopy and multivariate curve resolution

Shoichi Maeda[1,#], Shunta Chikami[1,#], Subin Song[1,#], Maria Vanessa Balois-Oguchi[2], Airi Katase[1], Glenn Villena Latag[1], Takuo Tanaka[2], and Tomohiro Hayashi[1,*]

[1]*Department of Materials Science and Engineering, School of Materials Science and Chemical Technology, Institute of Science Tokyo, 4259 Nagatsuta-cho, Midori-ku, Yokohama-shi, Kanagawa-ken 226-8502, Japan.*

[2] *Metaphotonics research team, RIKEN Center for Advanced Photonics, Hirosawa, Wako, Saitama 351-0198, Japan*

[#]equally contributed authors

[*]corresponding author: th@mct.isct.ac.jp

Contents:

Methodology of gap-controlled ATR-IR spectroscopy

- Spectral deconvolution using Multivariate Curve Resolution—Alternating Least Squares (MCR-ALS) method
- Calculation of thicknesses of interfacial regions
- A finite-difference time-domain (FDTD) calculation evaluating the electromagnetic field in the gap
- Validation of the sample-and-prism parallelism

Materials and calculations

- Fabrication of Self-Assembled Monolayers (SAMs)
- Preparation of a quartz surface
- Surface modification of polystyrene (PS) by UV-treatment
- Fabrication of polymer brush films
- FDTD Simulations

METHODOLOGY

Spectral deconvolution using Multivariate Curve Resolution—Alternating Least Squares (MCR-ALS) method

The MCR-ALS method was applied to deconvolute mixed spectra of bulk water and interfacial water using the set of spectra. The set of spectra is arranged in a data matrix **D** ($r \times c$). This matrix has r rows, corresponding to the number of spectra, and c columns, representing the number of wavenumbers. The MCR-ALS deconvolution of matrix **D** was performed using Equation (S1):

$$\mathbf{D} = \mathbf{C}\mathbf{S}^{\mathrm{T}} + \mathbf{E} \quad \text{(S1)}$$

where **C** ($r \times n$), $\mathbf{S}^{\mathrm{T}}$ ($n \times r$), and **E** ($r \times c$) are matrices representing the variations in contributions of n chemical species across different r rows of the data matrix, the variations in spectra of the n species, and the residual matrix containing data variance that cannot be explained by the product $\mathbf{C}\mathbf{S}^{\mathrm{T}}$, respectively. This relationship is illustrated in Figure S1.

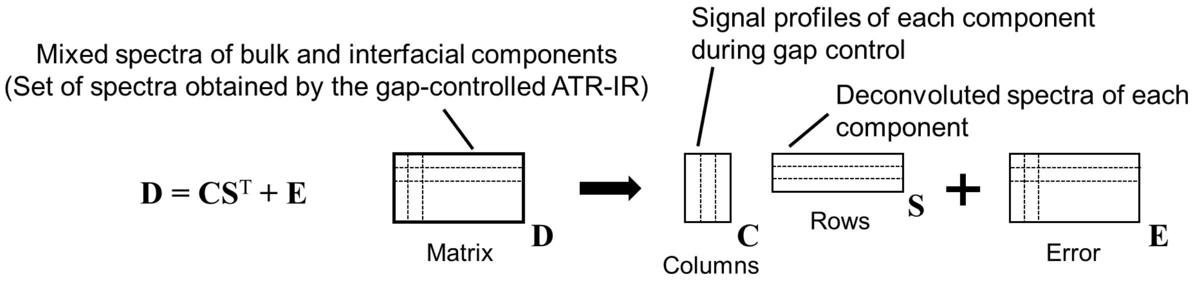

Figure S1. Principle of multivariate curve resolution (MCR) in this work.

The MCR-ALS analysis was conducted in two steps using a Python platform (version 3.8.5). The analysis focused on the OH stretching region (wavenumber range from 3800 to 2800 cm^{-1}) to extract the pure spectrum of interfacial water.

In the first MCR-ALS analysis, the experimentally obtained spectrum of bulk water served as the initial estimate for the bulk component, while the spectrum with the strongest signal from interfacial water, indicated by the lowest absorbance, functioned as the initial estimate for the interfacial component. During the MCR-ALS calculation, the initial estimate for the interfacial component was optimized while keeping the bulk water spectrum constant. The optimization process adhered to the constraint of non-negativity for both spectra.

Subsequently, difference spectra were obtained by subtracting each spectrum in the set of spectra $\mathbf{D}_1$. These were added to the set of spectra $\mathbf{D}_2$, as shown in Figure 4, to eliminate the contribution of interfacial water between the ATR prism and bulk water. As the interfacial water of the ATR prism does not change in signal intensity under the gap-control perturbation, its signal can be removed by obtaining difference spectra. The subtracted spectra served as the set of spectra for the second MCR-ALS analysis. Regarding the initial estimates for the second MCR-ALS analysis, the spectra of bulk water and interfacial water obtained by the first MCR-ALS analysis were used as the initial estimates for the bulk and interfacial components, respectively. This approach ensures a more accurate spectral deconvolution. Before performing the second MCR-ALS analysis, the raw spectrum of bulk water was replicated and added to the set of spectra $\mathbf{D}_2$ to equal the number of difference spectra. The procedure and results are presented in Figure S2.

The model adopted in this study showed a Lack of Fit (LOF) of less than 1%. The R-squared (R^2) value was greater than 0.99, indicating that over 99% of the total variance in the

data could be explained by the constructed model. Furthermore, the non-negativity of the spectral components was confirmed in the fitting.

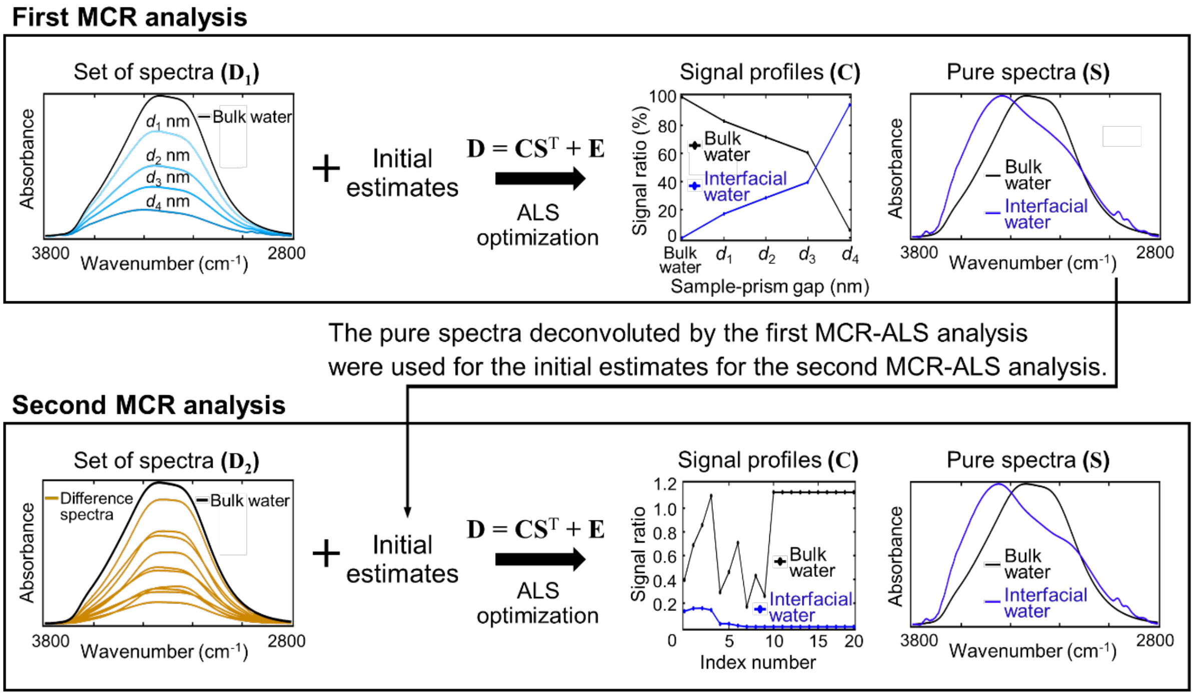

Figure S2. Flow of MCR-ALS analysis to deconvolute the mixed spectra of bulk and interfacial components using sets of spectra. (The index number in the second MCR-ALS analysis corresponds to the number of spectra in the set of spectra $\mathbf{D}_2$).

Calculation of thicknesses of interfacial regions

The analysis using MCR-ALS enables the calculation of the thicknesses of interfacial regions. By expressing d_p in terms of the wavenumber, d_p can be represented as Eq. (S2):

$$d_\mathrm{p} = \frac{1/\mathrm{wavenumber}}{2\,\pi\, n_1 \sqrt{\sin^2\theta - (n_2\,/\,n_1)^2}} = \frac{\mathrm{k}}{\mathrm{wavenumber}} \qquad \text{(S2)}$$

where k is a constant.

The total signal (i.e., the spectral area in the wavenumber range of 2800 to 3800 cm^{-1}) is obtained by summing the spectral intensities, I, at each wavenumber. The total signal from the bulk region can be expressed as Eq. (S3):

$$\text{Total signal}_{\text{of bulk region}} = \sum_{\text{wavenumber}=2800}^{3800} I \tag{S3}$$

The total signal when the sample-prism gap is d_1 can be expressed by Equation (S4) and illustrated in Figure S3(b):

$$\text{Total signal}_{\text{when gap} = d_1} = \sum_{\text{wavenumber}=2800}^{3800} \left\{1 - \exp\left(-\frac{2 \cdot d_1}{\text{k/wavenumber}}\right)\right\} \tag{S4}$$

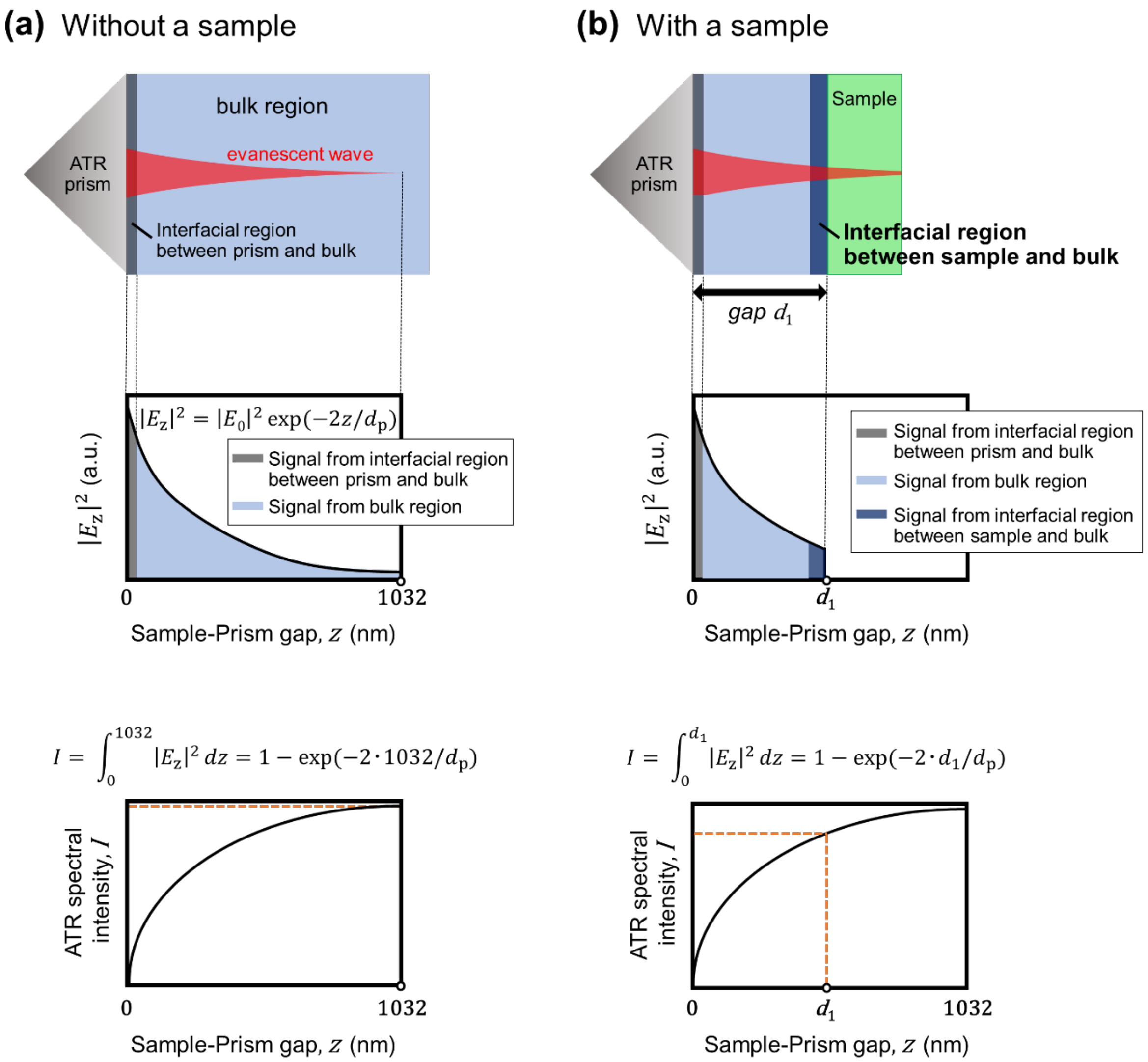

Figure S3. Spectral intensity (at a wavenumber of 3300 cm^{-1}) of molecules in contact with the prism as a function of the sample-prism gap for systems (a) with and (b) without a sample. Here, d_1 represents the sample-prism gap in (b). Note that the signal from the interfacial region of the ATR prism (gray) is consistently present in both cases.

Additionally, the total signals of (a) and (b) in Fig. S3 can be obtained not only by using Equations (S3) and (S4) but also by calculating the areas of the experimentally obtained spectra, which offers a relationship given in Eq. (S5).

$$\frac{\sum_{\mathrm{wavenumber}=2800}^{3800}\left\{1-\exp\left(-\frac{2\cdot d_1}{\mathrm{k/wavenumber}}\right)\right\}}{\sum_{\mathrm{wavenumber}=2800}^{3800}\left\{1-\exp\left(-\frac{2\cdot\infty}{\mathrm{k/wavenumber}}\right)\right\}}=\frac{\text{Spectral area}_{\text{ when gap }=\, d_1}}{\text{Spectral area}_{\text{ of bulk region}}} \tag{S5}$$

By calculating Eq. (S5), the sample-prism gap, d_1, can be determined. By performing the same calculation, all sample-prism gaps can be determined. The gaps between C8-SAM and the prism are calculated as follows:

The thickness of the interfacial region (as shown by "T" in Fig. S4) was determined using Eq. (9). By subtracting the spectrum at gap d_2 from that at gap d_1, the signal from the interfacial region between the prism and bulk is eliminated, resulting in a difference spectrum that includes only the signals from the bulk and interfacial regions. The signal ratio between the bulk and interfacial components can be expressed by Eq. (S6), which is illustrated in Fig. S4.

$$\text{Signal ratio}\left(\frac{\text{Interfacial region}}{\text{Bulk region}}\right)=\frac{\int_{d_2-T}^{d_2}|E_z|^2\,\mathrm{dz}-\int_{d_1-T}^{d_1}|E_z|^2\,\mathrm{dz}}{\int_{d_2-T}^{d_1-T}|E_z|^2\,\mathrm{dz}} \tag{S6}$$

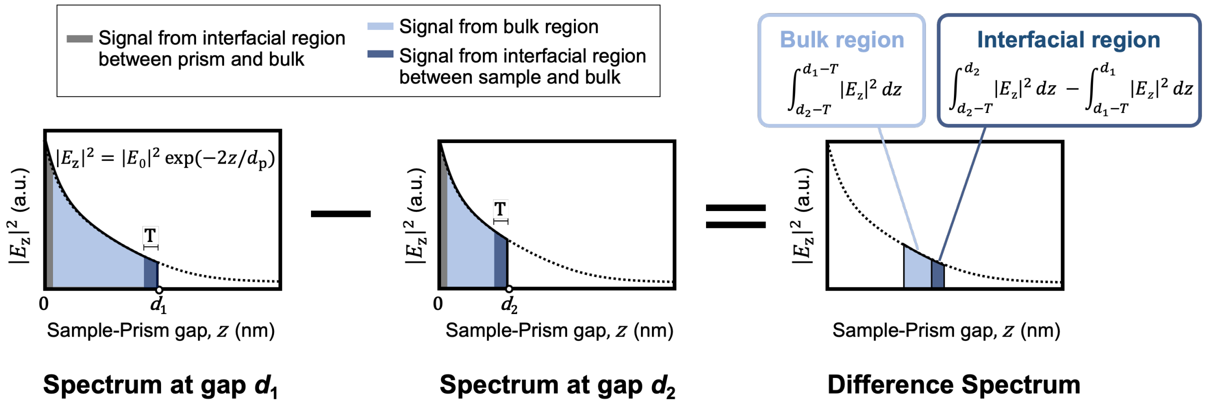

Figure S4. The spectral component of the difference spectrum in the OH stretching region obtained by subtracting the spectrum at gap d_2 from the spectrum at gap d_1. T represents the

thickness of the interfacial region. It is important to note that the difference spectrum does not include the signal from the interfacial region between the prism and the bulk region.

Furthermore, the signal ratio between the bulk and interfacial regions can also be obtained from the concentration profile in the second MCR-ALS analysis, resulting in the relationship expressed by Eq. (S6) and described using Eq. (S7):

$$\text{Signal ratio obtained by MCR}-\text{ALS}\left(\frac{\text{Interfacial region}}{\text{Bulk region}}\right) = \frac{\int_{d_2-T}^{d_2} |E_z|^2\,dz - \int_{d_1-T}^{d_1} |E_z|^2\,dz}{\int_{d_2-T}^{d_1-T} |E_z|^2\,dz} \quad (S7)$$

From Eq. (S7), the *T* value, representing the thickness of the interfacial region, can be calculated. This thickness can be determined by performing the calculation for all difference spectra and then averaging the values of *T*.

Finite-difference time-domain (FDTD) calculation evaluating the electromagnetic field in the gap

We calculated the distribution of the electromagnetic (EM) field intensity in the gap using the finite-difference time-domain (FDTD) method because the reflection of the evanescent field on the sample causes the intensity to deviate from the theoretically predicted exponentially decaying profile (Eq. 1). Fig. S5 displays the intensity of the EM field in the gap as a function of the distance from the prism surface at varying prism-sample distances. At any gap size, the intensity of the EM field exhibits exponential decay. However, when the gap is smaller than 100 nm, the effect of reflection on the EM field intensity becomes significant. At a gap size of 100 nm, the intensity substantially depends on the prism-sample distance. Therefore, we evaluated the spectra of the interfacial region and the thickness of the interfacial region using data obtained at a gap distance greater than 300 nm.

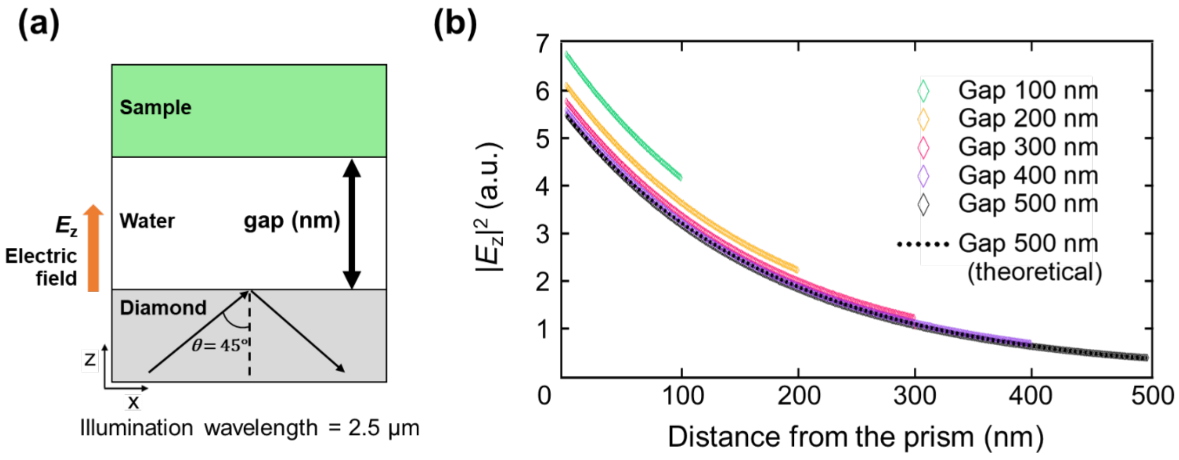

Figure S5. (a) Model used for the FDTD calculation; (b) the square of the electric field intensity as a function of the sample-prism gap obtained from FDTD (with PDMS for the sample). The calculations were performed with various prism-sample distances. The figure also illustrates the curve derived from the theoretical equation.

Figure S6 illustrates the intensity of the EM field plotted as a function of the distance from the prism surface (z). The dotted lines represent the theoretical decay curve based on Eq. 1, assuming the same intensity of $|Ez|^2$ at $z = 0$. When comparing Au, quartz, and PDMS, the EM field between Au and the prism is stronger than in the other two cases, indicating that the reflection of the evanescent field is significant for Au surfaces. This effect leads to an overestimation of the thickness of the interfacial region, T. Therefore, we calibrated the value of T by using the ratio of theoretical to calculated values of $|Ez|^2$ in the case of Au samples.

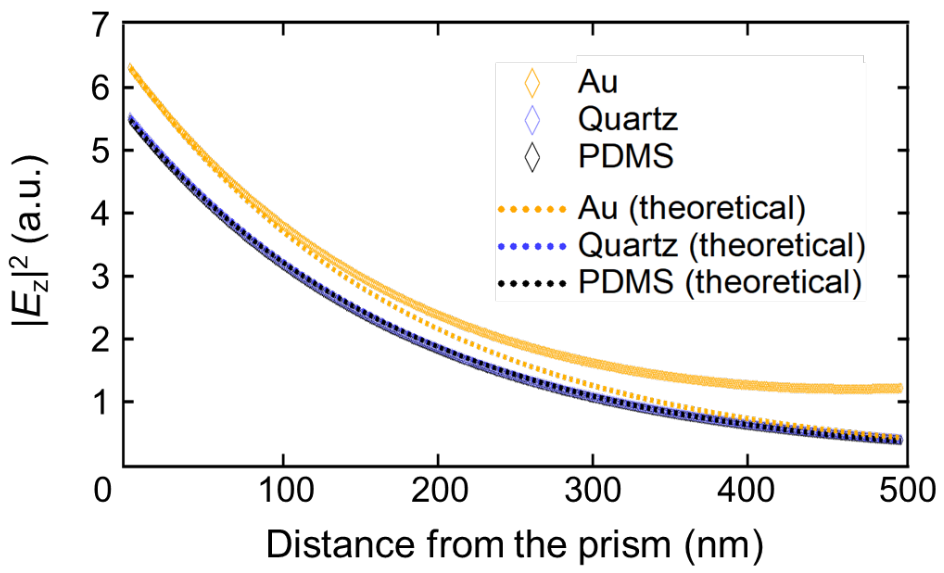

Figure S6. The calculated EM field as a function of the distance from the prism.

Validation of the sample and prism parallelism

The parallelism of a sample and prism is essential for evaluating the thickness of the interfacial region. In most cases, the parallelism is maintained using a spacer of uniform thickness. However, parallelism is not achieved in some instances. In such cases, we can detect the tilting of the sample relative to the prism by monitoring the ratio between the intensities of the signals from the sample and the confined liquid. The relationship between the signals will follow Eq. (S8) (bulk material) and Eq. (S9) (for a thin film with a thickness of 1 nm), where A, A′ , B, and B′ are constants. The relationships are plotted in Fig. S7. These relationships were obtained by integrating the theoretical signals based on the system's configuration. When the sample surface is not parallel, the relationship deviates from the theoretical trend (Fig. S7). In most cases, we achieve parallelism by adjusting the spacer and the positions of the sample.

$$\text{CH/OH peak intensity ratio of bulk material} = \frac{\int_d^{1030} \mathrm{A} \exp(-2z/499)\, \mathrm{d}z}{\int_0^d \mathrm{A'} \exp(-2z/448)\, \mathrm{d}z} \quad \text{(S8)}$$

$$\text{CH/OH peak intensity ratio of thin film} = \frac{\int_d^{d+1} \mathrm{B} \exp(-2z/499)\, \mathrm{d}z}{\int_0^d \mathrm{B'} \exp(-2z/448)\, \mathrm{d}z} \quad \text{(S9)}$$

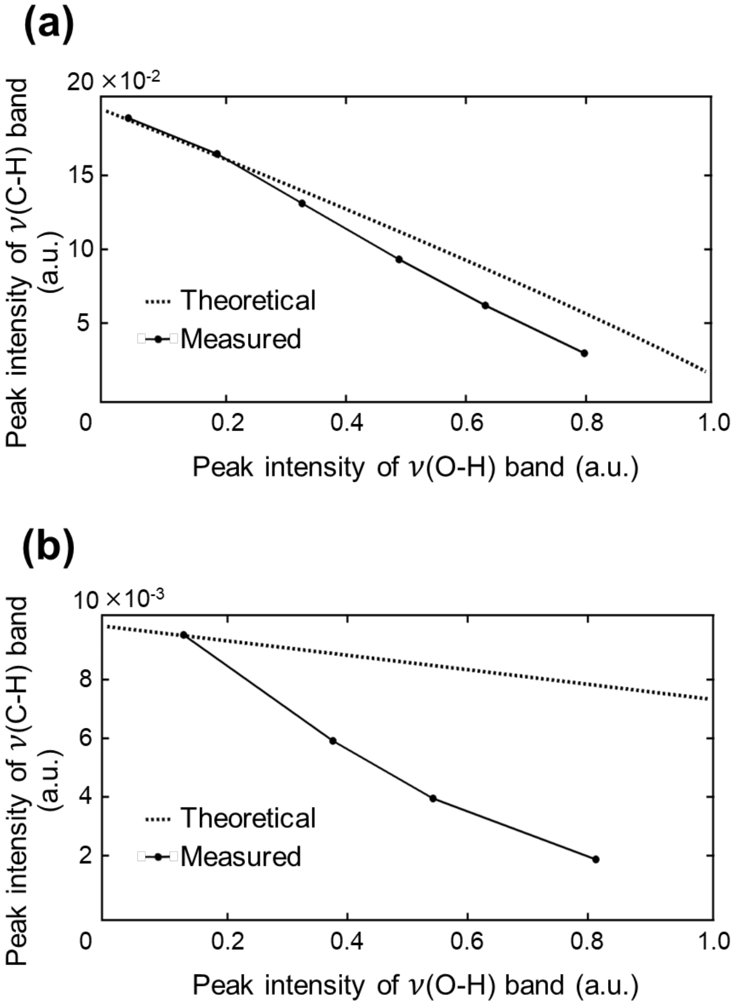

Figure S7. Peak intensity of ν(C-H) stretching modes (~ 2960 cm^{-1}) of (a) PDMS (bulk material) and (b) C8 SAM (thin film) plotted as a function of the peak intensity of the confined liquid (the OH stretching peak of the water in this case) (~ 3300 cm^{-1}) when the sample surfaces are not parallel to the prism.

Materials

PDMS films were prepared using a PDMS mixture that contains a 10:1 weight ratio of a base compound and a curing agent (SILPOT 184 W/C, Dow Corning Toray Co., Ltd.). The mixture was coated onto a silicon substrate, which served as a template (silicon wafer, p-type, [100], RMS roughness is less than 2 nm), and degassed in a vacuum desiccator for 1 h. It was then cured in an oven at 70°C for approximately 3 h, followed by overnight storage to complete the preparation. The RMS roughness of the resulting PDMS film was about 3-5 nm.

Fabrication of Self-Assembled Monolayers (SAMs)

Au (111) substrates were prepared by depositing gold onto freshly cleaved mica (10 × 10 × 0.3 mm, S & J TRADING INC.) at 700 K, under a vacuum pressure of 10^{-5}-10^{-6} Pa. The substrates were subsequently annealed at 700 K in a vacuum chamber for 5h. Three types of SAMs were fabricated by immersing the Au (111) in freshly prepared ethanolic solutions, each containing the corresponding compound at a concentration of 1 mM, for 24 h, as listed in Table S1. Following immersion, the samples were carefully rinsed with pure ethanol to remove physisorbed thiol molecules from the surface.

Table S1. List of derivatives of alkanethiols used in this work.

Abbreviation	Chemical structure of the thiol molecules	Static water contact angle (°)
C8[a]	$HS\text{-}(CH_2)_7\text{-}CH_3$	112 (3.1)
OH[a]	$HS\text{-}(CH_2)_{11}\text{-}OH$	17 (2.6)
EG3-OH[b]	$HS\text{-}(CH_2)_{11}\text{-}(OCH_2CH_2)_3\text{-}OH$	32 (4.3)

Purchased from [a]Sigma-Aldrich and [b]ProChimia Surfaces. Numbers in parentheses are standard deviations. $N = 5$.

Preparation of a quartz surface

Three types of surfaces were studied: non-treated, protonated, and deprotonated quartz substrates. Smooth quartz slides (15 × 15 × 1 mm, KENIS, Ltd.) were cut into pieces measuring 10 × 10 mm^2 to serve as substrates. The quartz substrates underwent ultrasonic cleaning in acetone, ethanol, and pure water, followed by a nitrogen gas blow. The pH of each solution was adjusted by adding the appropriate amounts of HCl (Nacalai Tesque, Inc., >99.5%) or NaOH (Merck, 99.99%) solutions.

Surface modification of polystyrene (PS) by UV-treatment

Two types of surfaces were studied: untreated and plasma-treated PS petri dishes (AS ONE Corporation). The untreated PS petri dish was thoroughly washed with pure water and subsequently dried using nitrogen gas. The plasma-treated PS petri dish was prepared by plasma irradiation for 10 min.

Fabrication of polymer brush films

Polymer brush films were synthesized using the “grafting-to” technique, employing poly(CBMA) (Nicca Chemical, Fukui, Japan), and thiol-modified polyethylene glycol (PEG) (NOF Corporation, Japan) were purchased and purified through dialysis (Float-A-Lyzer G2 Dialysis Device, MWCO 3.5-5kD, Spectrum Laboratories Inc., NJ, U.S.).[2]

Surface plasmon resonance (SPR) chips (Cytiva, Marlborough, U.S.) were initialy cleaned and initiator SAM was subsequently formed on the chips. Polymer brush films were then fabricated on the SPR chips. The methods for these procedures are detailed in our previous

work.[2] The chemical structures of CBMA and EG, which constitute the polymer brush films, are presented in Fig S8.

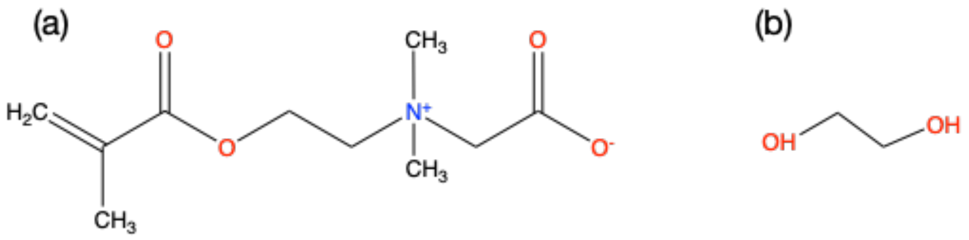

Figure S8. Chemical structures of (a) Carboxybetaine methacrylate (CBMA) and (b) Ethylene glycol (EG)

FDTD Simulations

The FDTD simulations were conducted using a commercially available software (Ansys Lumerical). The model used is shown in Fig. S5(a). It consists of a three-layer system with the bottommost to topmost layers being: diamond, water gap, and PDMS, respectively. The gap size used during the simulations were 100 nm, 200 nm, 300 nm, 400 nm, and 500 nm. The incident electric field, with a wavelength of 2.5 μm, was linearly polarized parallel to the plane of incidence (p-polarization) in Fig. S5 and polarized perpendicular to the plane of incidence (s-polarization) in Fig. S5 (for $|Ey|^2$). The propagating electric field entered the three-layer system through the diamond layer at an incidence angle of 45°. The three-dimensional model was enclosed in a FDTD space spanning 1000 nm in both the *x*- and *y*-directions and 6000 nm in the *z*-direction. Bloch boundary conditions were used in the *x*- and *y*-directions, while perfectly matched layers were used in the *z*-direction. The mesh size used in the *z*-direction was 1 nm while a non-uniform mesh was used in the *x* and *y* directions to minimize the required memory and computation time for the simulations. The dielectric constants used for each

material (at λ = 2.5 μm) were: ε_1 = 5.66814 for diamond[3], ε_1 = 1.921 for PDMS[4] and ε_1 = 1.58733, ε_2 = 0.00526844 for water[5].

References

(1) Woods, D. A.; Bain, C. D. Total Internal Reflection Spectroscopy for Studying Soft Matter. *Soft Matter* **2014**, *10*, 1071–1096.

(2) Palai, D.; Tahara, H.; Chikami, S.; Latag, G. V.; Maeda, S.; Komura, C.; Kurioka, H.; Hayashi, T. Prediction of Serum Adsorption onto Polymer Brush Films by Machine Learning. *ACS Biomater. Sci. Eng.* **2022**, *8*, 3765–3772.

(3) Phillip, H. R.; Taft, E.A. Kramers-Kronig Analysis of Reflectance Data for Diamond. Phys. Rev. 1964, 136, A1445-A1448.

(4) Querry, M. R. Optical Constants of Minerals and other Materials from the Millimeter to the Ultraviolet, Contractor Report 1987, CRDEC-CR-88009.

(5) Hale, G. M.; Querry, M. R. Optical Constants of Water in the 200-nm to 200-μm Wavelength Region, Appl. Opt. 1973, 12, 555-563.
